# Path Analysis Association between Domestic Violence, Anxiety, Depression and Perceived Stress in Mothers and Children’s Development

**Published:** 2016

**Authors:** Roshanak VAMEGHI, Sedigheh AMIR ALI AKBARI, Firoozeh SAJEDI, Homeira SAJJADI, Hamid ALAVI MAJD

**Affiliations:** 1 Pediatrician, Pediatric Neurorehabilitation Research Center, University of Social Welfare and Rehabilitation Sciences. Tehran, Iran; 2 PhD Candidate, Pediatric Neurorehabilitation Research Center, University of Social Welfare and Rehabilitation Sciences. Tehran, Iran; 3 Community Medicine, Social Determinants of Health Research Center, University of Social Welfare and Rehabilitation Sciences, Tehran, Iran; 4 Biostatistician, Department of Biostatistics, School of Paramedical Sciences, Shahid Beheshti University of Medical Sciences, Tehran, Iran

**Keywords:** Domestic violence, Depression, Anxiety, perceived stress, Developmental delay, Path analysis

## Abstract

**Objective:**

Given that several factors involved in the incidence or exacerbation of developmental disorders in children, the present study aimed to investigate the relationship between some of the risk factors affecting mothers’ health and development in children using path analysis.

**Materials & Methods:**

The present cross-sectional analytical study was conducted on 750 mothers and their children in health centers in Tehran, Iran in 2014 enrolled through multi-stage random sampling. Data were collected using a demographic and personal information questionnaire, the Perceived Stress Scale, Beck’s depression Inventory, Spielberger’ anxiety inventory, the WHO domestic violence questionnaire and an ages & stages questionnaire for assessing children’s development. Data were analyzed using SPSS.19 (Chicago, IL, USA) and Lisrel 8.8.

**Results:**

Developmental delay was observed in 12.1% of the children. The mean stress score was 23.94±8.62 in the mothers, 50.7% of whom showed mild to severe depression, 84.2% moderate to severe anxiety and 35.3% had been subjected to domestic violence. The path analysis showed that children’s development was affected directly by perceived stress (β=-0.09) and depression (β=-0.17) and indirectly by domestic violence (β=-0.05278) and anxiety (β=-0.0357). Of all the variables examined, depression had the biggest influence on development in the children (β=-0.17). The proposed model showed a good fit (GFI=1, RMSEA=0.034).

**Conclusion:**

Children’s development was influenced indirectly by domestic violence and anxiety and directly by perceived stress and depression in mothers. It is thus suggested that more concern and attention be paid to women’s mental health and the domestic violence they experience.

## Introduction

Developmental delay in children is a major pediatric health issue in developing and developed countries (1, 2). Developmental delay is referred to a child whose prominent developmental features are not appropriate for his age (3). According to statistics, 200 million children under the age of 5 years suffer from this condition and do not reach their potential level of development (1). The level of developmental delay varies across countries. The prevalence of developmental disorders has been 7.1% to 26% in India (4, 5), 44.6% in Ghana (6), 46.1% in Colombia (7), 31.7% in Brazil (8) and 46% in Canada (9). In the US, developmental delays have been reported from 3.4% to 16% in different domains and nearly 30% of these disorders are not detected before school age (10, 11). In Iran, developmental delay in various domains has been reported from 3.69% to 4.31% in Tehran (12) and 11.8% to 30% in other cities in different age groups (13-16). The special needs of these children in terms of detection, treatment, care and education usually create difficulties for their families, the health system, and the education system (13). 

In addition to biological factors such as malnutrition, iron deficiency, anemia and low birth weight, several factors play a role in a child’s development; the effect of environmental and social factors on the child’s neurodevelopment cannot be overlooked either (17). A link between high-risk psychosocial factors, such as poverty, single parenting and the mothers’ poor education and general and mental health, and developmental disorders and delays in children is suggested (18).

There are a relatively large number of studies on the relationship between children’s development and mothers’ mental health and psychosocial harms such as stress, anxiety, depression and domestic violence (13, 19-22). Children living with mothers who have psychological problems are deprived of proper motor, cognitive and psychosocial development (23, 24). Stress is a major risk factor in life defined as a physiological response to physical and psychological needs and threats (25). Evidence also suggests a strong relationship between stressful events in women and developmental delays in their children. Mental health problems in parents and the resultant stress are linked to children’s behavioral, mental and emotional problems (26, 27). Children living with mothers who experience stressful circumstances have learning difficulties due to receiving inadequate learning stimulation and are deprived of an adequate quality of care due to the insufficient attention they receive from their mother, creating severely irritable children with behavioral problems (28, 29).

Maternal depression is one of the factors that affect children’s development. Depressed mothers find it difficult to relate to their children. Previous studies have shown a relationship between maternal depression and children’s cognitive development (19, 20). A relationship has been suggested between children’s language, gross motor and emotional development and maternal depression (21). Depressed mothers are often unable to meet their children’s social and emotional needs, creating a series of limitations for the mother and a failure to perform her maternal duties and thus leading to cognitive problems in the mother and a negative recognition of and function toward the child and other family members and provoking further behavioral problems in the child (20).

Maternal anxiety is another psychological factor that affects children. Anxiety is caused by the exposure to actual or perceived stressful situations (30). Prenatal anxiety has been reported as a predictor of disorders such as attention deficit disorders and hyperactivity in children. These children have experienced additional psychological and emotional problems (31). Highly anxious mothers are more likely to have hyperactive and irritable children (32). 

Domestic violence is another problem affecting women’s mental health. Domestic violence is taken to refer to physical, sexual, mental or emotional violence inflicted on the woman by her spouse or relatives (33) and constitutes a major health challenge, even in developed countries (34). At least 30% of women are subjected to domestic violence and experience high levels of anxiety, stress and depression (33). In Brazil, 72% of women subjected to domestic violence suffered from depression (35). Of the women subjected to sexual violence by their partner, 41% reported depression, 23% reported anxiety and 12% reported stress (36). Mothers subjected to violence fail to properly perform their maternal role and thus raise children with behavioral and emotional problems (37). Psychological problems experienced by the mother due to domestic violence impair her performance of the maternal role and negatively affect her intimacy with the child and cause aggressive behaviors in her toward the child, thereby threatening the physical and psychological health of the child as well. Domestic violence has been proposed as an indirect factor contributing to children’s behavioral problems such as aggression (38, 39).

Given that several factors are involved in the incidence or exacerbation of developmental disorders in children, and since the mother’s health affects the child’s health, and also given the scarcity of domestic studies on this subject, especially studies that analyze a combination of factors in interaction with each other and as a path, the present study aimed to investigate the relationship between multiple risk factors affecting mothers’ health, including domestic violence, anxiety, depression and perceived stress, and development in 6 to 18 month-old children using a path analysis. 

## Materials & Methods

The present cross-sectional path analysis was conducted on 750 mothers and their 6 to 18 month-old children in health centers located in different areas of Tehran, Iran, in 2014.

**Fig 1:**
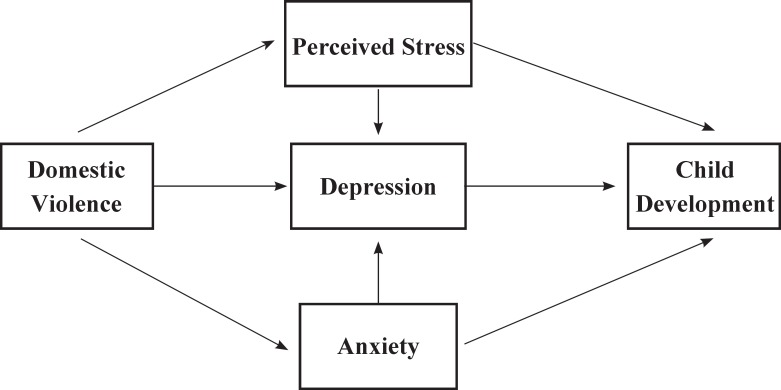
The Oretical path model for effects of domestic violence, perceived stress, depression and anxiety in mothers on child development

The initial design for the proposed communication model was developed through a search for resources and evidence in databases such as PubMed, Scopus, SID and Google Scholar using key words such as psychological factors, psychological disorders, domestic violence, stress, perceived stress, depression, mother, child development and developmental delay both individually and in combination. Through an extensive in-depth review of the different articles retrieved and a contemplation of personal experiences, the research team proposed the initial communication model (Figure 1). This model was reviewed and confirmed by another group of maternal and infant health professionals with no connections to the project. Taking into account the 20% prevalence of developmental delays and the study variables, the minimum sample size was determined as 700 and 750 mothers entered the study along with their children. The study was approved by the Ethics Committee of the University of Social Rehabilitation and Welfare Sciences and sampling began after obtaining the necessary permissions from the university and the Health Deputy of the Ministry of Health and Medical Education. Multistage sampling was carried out by identifying the municipal northern districts of Tehran. A list was then prepared of all the healthcare clinics in these districts (as clusters) and some were randomly selected from each district. A share of the samples was then dedicated to each clinic based on the population it covered. Iranian mothers aged 18 to 35 yr who had breastfeeding infants and a parity of less than four and had no current or prenatal history of known medical diseases such as diabetes and cardiovascular, kidney, pulmonary or autoimmune diseases entered the study. 

Moreover, their infants had to have been born as a full-term singleton with an Apgar score above 7 and a minimum birth weight of 2500 grams. The infants had received iron supplements during the first six months of life and their physical health was confirmed by the clinic physician before entering the study. 

Mothers with pregnancy complications such as placental abruption, pre-eclampsia and polyhydramnious and whose recent childbirth had been prolonged or had required the use of forceps or vacuum and had been accompanied by complications such as bleeding and dystocia were excluded from the study. Having children with developmental disorders and known congenital abnormalities on either the mother’s or the father’s side of the family indicated exclusion from the study. Infants with growth restriction and previous hospitalization were also excluded. 

Data were collected using a demographic and personal information questionnaire for the parents and the children, the Perceived Stress Scale, Beck’s Depression Inventory, Spielberger’ Anxiety Inventory, the Domestic Violence Questionnaire and an Ages & Stages Questionnaire for assessing children’s development. The questionnaire on the parents’ demographic information, the mother’s obstetrics history and the child’s personal information was researcher-designed and had validity confirmed by tenfaculty members through the content validity method. 

The Ages & Stages is a development assessment tool for children aged 4 to 60 months and consists of 19 questionnaires. The sum of the scores obtained in five areas is compared against the standard scores (cut-off points). The child’s failure to reach the cut-off point in any of the five areas indicates problems in that area.

Cut-off points were determined for Iranian children according to the study conducted earlier (12). The Ages & Stages questionnaire had been standardized for Iranian children already (40)., and different studies have reported the sensitivity of the items for different age groups to range between 0.7 and 0.9, its specificity to be between 0.76 and 0.91 (moderate to good) and its internal consistency with Cronbach’s alpha to range from 0.76 to 0.86 and its test-retest reliability to be 0.93(40-43). This questionnaire is currently used in all university-affiliated clinics across the country based on the guidelines provided by the Ministry of Health and Medical Education for assessing children. 

The present study found the test-retest reliability of this questionnaire between 0.95 and 0.98 in the four different age groups.

The perceived stress questionnaire was developed by Cohen et al. in 1983 and is used to assess general perceived stress over the preceding month through assessing personal thoughts and feelings about the stressful events and mental pressures experienced and the degree of control over them and the coping and coming to terms with them (44). This questionnaire has been translated into different languages widely used and standardized in different countries. The 14-item version of the questionnaire was used in the present study and participants had to answer the items based on a 5-point Likert scale from “never” given a score of zero to “very often” given a score of five; the total score obtained then ranges from 0 to 56 and higher scores indicate higher perceived stress, and no cut-off points were thus determined for the questionnaire. The reliability of the Persian version of the questionnaire was determined by Bastani et al. through measuring its internal consistency with a Cronbach’s alpha of 0.74 (45). Other domestics’ studies have reported its Cronbach’s alpha as 0.84 to 0.86 (46-49). The internal reliability of the questionnaire was reported as 0.86 and its test-retest reliability as 0.89 in the present study. 

Spielberger’s Anxiety Inventory contains two groups of questions, including 20 items on state anxiety and 20 items on trait anxiety measuring the severity of the symptoms. Answers are given a score from 1 to 4, making the lowest score 20 and the highest 80. A score of 20 to 40 indicates mild anxiety, 41 to 60 moderate anxiety and 61 to 80 severe anxiety. Spielberger’s Anxiety Inventory is a valid questionnaire whose reliability has been determined in several studies. In Iran, the reliability of the inventory was determined in studies conducted in Tehran (0.91) and Mashhad (0.95) (50-53). The present study calculated the internal reliability of the inventory as 0.85 and its test-retest reliability as 0.87. 

Beck’s Depression Inventory contains 21 items and is given a score ranging from 0 to 63. A score of 0 to 9 indicates a normal level of depression, 10 to 18 indicates mild depression, 19 to 30 moderate, 30 to 40 severe and 41 to 63 very severe depression. Various studies have confirmed the reliability of this inventory, which has also been standardized for use in Iran with 9 as its cut-off point for the Iranian population. Studies reported the internal validity of the inventory with a Cronbach’s alpha of 0.87 and its reliability as 0.74 (54- 56). The present study calculated the internal reliability of the inventory as 0.89 and its test-retest reliability as 0.92.

The present study considered domestic violence as the experience of violence by the spouse measured through a questionnaire developed by the WHO in physical (9 items), sexual (8 items) and emotional (15 items) domains. The instances of domestic violence experienced by the respondent are scored based on a 5-point Likert scale. One positive answer to any of the items in the physical, sexual, or emotional domains indicates a wo man who is subjected to violence. This questionnaire has been validated by many researchers in Iran and the Cronbach’s alpha values calculated for its physical, emotional and sexual domains were reported as 0.92, 0.89 and 0.88, in respective order (57). 

The present study calculated the internal reliability of the questionnaire as 0.92 and its test-retest reliability as 0.94. 

After briefing them on the study objectives and obtaining written consents, the questionnaires were distributed among the mothers. Research ethics were observed by referring the children with unfavorable scores to the clinic physician and the mothers with psychological problems to the relevant clinic department. 

The initial model proposed was evidence-based Figure 1) and was tested in Lisrel8.8 for the path analysis. Path analysis is an extension of regular regression analysis that is able to show both the direct and indirect effects and the overall effect of each of the study variables on the dependent variables and to interpret the relationships and correlations observed between them in a logical way. Path analysis shows the more important or significant path of the proposed model. The path coefficients are calculated based on the standardized regression coefficient. Each variable is assumed a function of the other variables and its regression model is plotted. To obtain estimates of the main path coefficients, it suffices to regress each (endogenous) dependent variable on those variables that directly impinge on it. In other words, standardized regression coefficients (path coefficients) are calculated for obtaining the estimates of each identified path. 

These coefficients are calculated through structural equations, i.e. equations that identify the structure of the assumed relationships in a model (58). 

In this method, the overall effect of a variable on another variable is calculated by adding its “direct effect” and “total indirect effects”. The RMSEA, Goodness of Fit Index (GFI), Normed Fit Index (NFI) and Comparative Fit Index (CFI) are used in the present study to determine the fit of the model.

Data were analyzed in LISREL and SPSS-19 (Chicago, IL, USA) using the Mann-Whitney test, the Chi-square and the independent t-test at a significance level of 0.05.

## Results

The mean age of the 750 children studied was 10.53±4.46 months, and 29.6%, 23.7%, 25.6% and 21.1% were at the age group of 6-8-12 and 18 months, respectively. As for the gender of the children, 48% were girls. A total of 12.1% of the children showed developmental delays. No significant differences were observed between the group of children with normal development and the group with delayed development in terms of their parents’ mean age, education and family income (Table1). 98.3% of the mothers in the group of children with normal development and 98.6% of those in the group of children with delayed development were housewives, constituting no statistically significant differences between the groups in this regard. The Chisquare test showed no significant differences between the two groups of children in terms of the type of delivery and the planned or unplanned nature of the pregnancy according to the parents.

**Table 1:** Comparison of the Parents’ Demographic Features by Their Child’s Developmental Status in Cases Referred to Health Centers Affiliated to Shahid Beheshti University of Medical Sciences in 2014

**Groups**		**Normal development** **N=659**	**Delayed Development** **N=91**	**Results**
**Variables**				
**Mean &SD of Mother age**		29.09±5.06	29.34±5.44	p=0.659
**Mean &SD of Father age**		33.34±5.47	33.63±5.43	p=0.643
**Level of Mother education** **Frequency(percent)**	**Primary**	64(9.7)	8(8.8)	p=0.131
	**High school**	391(59.3)	52(57.1)	
	**Diploma**	167(25.4)	26(28.6)	
	**College**	37(5.6)	5(5.5)	
**Level of Father education** **Frequency(percent)**	**Primary**	63(9.5)	6(6.6)	p=0.375
	**High school**	407(61.8)	56(61.5)	
	**Diploma**	147(22.3)	20(22)	
	**College**	42(6.4)	9(9.9)	
**Mean &SD of Family income per mouth** **(×10000=Million Rials)( Iran Currency =Rials)**		1062.89±331.21	1069.78±371.62	p=0/923

The highest frequency of delayed development was observed in the 8 month-old age group and the most frequent area in which the delays occurred was problem-solving (Table 2).

**Table 2:** Children’s Developmental Status by Age Group and Domain of Development

**Groups**		**Normal development**	**Deleyed development**
**Children age ** **(months)**		**Frequency(percent)** **659(87.9)**	**Frequency(percent) ** **91(12.1)**
6 (N=222)		197(26.3)	25(3.3)
8 (N=178)		147(19.6)	31(4.1)
12 (N=192)		164(21.9)	28(3.7)
18 (N=158)		151(20.1)	7(0.9)
**Total** **(N=750)**	**Domains of development**	**Frequency (percent)**	**Frequency (percent)**
	**Communication**	730(97.3)	20(2.7)
	**Gross motor**	719(95.9)	31(4.1)
	**Fine motor**	729(97.2)	21(2.8)
	**Problem solving**	690(92)	60(8)
	**Personal-social**	714(95.2)	36(4.8)

The mean stress score obtained by the mothers was 23.94±8.62, indicating moderate to high levels of stress; 50.7% of the mothers showed mild to severe degrees of depression, 84.2% had moderate to severe anxiety and 35.3% had been subjected to violence. Comparison between ASQ score, Maternal Depression, Domestic violence Perceived stress and Anxiety in two groups of children showed in Table 3.

**Table 3:** Comparison between ASQ Score, Maternal Depression, Domestic violence Perceived Stress and Anxiety in Two Groups of Children

**Groups**	**Normal development** **N=659**	**Delayed development** **N=91**	**Results**
**Variables**	**(Mean±SD)**	**(Mean±SD)**	
**ASQ score**	271.83±21.96	215.65±37.42	p<0.001
**Maternal Depression**	10.95±9.53	14.44±9.58	P<0.001
**Maternal** ** Domestic violence**	4.17±1.98	4.97±2.38	p=0.411
**Maternal Perceived stress**	22.94±8.47	29.55±4.85	P<0.001
**Maternal Anxiety**	43.82±5.62	44.65±5.85	p=0.192

**Fig 2:**
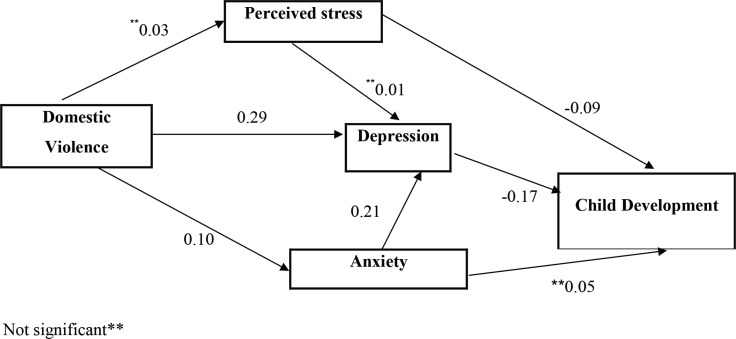
Full empirical model [(empirical path model for the effects of domestic violence, perceived stress, depression and anxiety in mothers on child development (β standardized was showed in model)]

In testing the communication model, a significant relationship was observed between domestic violence experienced by the mother and the incidence of depression and anxiety. In other words, depression and anxiety increased with the score obtained on domestic violence; however, the relationship with stress was not significant. The depression score also increased with the level of anxiety; however, no relationships were observed between an increased level of stress and the depression score. The model testing ultimately showed a significant inverse relationship between the mother’s stress and depression and her child’s development, as the child’s development score lowered with an increase in the mother’s level of depression and anxiety (Figure 2).

The results obtained showed that children’s development is directly and negatively affected by depression and perceived stress and indirectly and negatively by domestic violence and anxiety. In comparing the overall effects of the study variables, depression was found to have the biggest influence on children’s development (Table 4).

**Table 4:** Path Coefficients for Domestic Violence, Perceived stress, Depression and Anxiety in Mothers on Child Development

**Predictor Variables**	**Effect ( β standardized)**			**T-Value**
	**Direct**	**Indirect**	**Total**	
**Domestic Violence**	-	0.05287	0.05287	-
**Depression**	-0.17	-	-0.17	4.62
**Perceived Stress**	-0.09	-	-0.09	2.67
**Anxiety**		-0.357	-0.357	-

The indices used showed that the proposed model has a good fit (Table 5).

**Table 5:** Goodness of Fit Indices for the Model

χ2	df	p	NFI	CFI	GFI	RMSEA
3.78	2	0.15	0.98	0.99	1	0.034

## Discussion

The results obtained showed that children’s development is directly and negatively affected by depression and perceived stress and indirectly and negatively by domestic violence and anxiety. In comparing the overall effects of the study variables, depression was found to have the biggest influence on children’s development (Table 4).

The examined children showed a developmental delay of 12.1%, with the most frequent delay occurring in the area of problem solving and the least frequent in the area of communication. In one study, the frequency of developmental delay in different areas was reported as 3.69% to 4.31% in Tehran (12), with the most frequent delay occurring in the domain of fine motor skills and the least frequent in the domain of personalsocial development. The disparity of results between the present study and the one by Sajedi et al. may be attributed to the present research being set in Tehran and having examined children younger than 18 months, while the other study was conducted across the entire country on children from a wider age range; the study year and the effect of time and the particular social conditions of the society on children’s development may have also contributed to the disparity of findings.

The path analysis of depression and perceived stress affected children’s development directly while domestic violence and anxiety affected it indirectly, and depression was the mediating factor in both cases. 

In comparing the overall effects, depression had the biggest influence on children’s development. Domestic violence, anxiety and depression in mothers were linked.

The present study confirmed the indirect effect of domestic violence on children’s development and the relationship between domestic violence and depression in mothers. A relationship between domestic violence and depression in women is found (59, 60). Other studies have also demonstrated the indirect role of domestic violence against mothers in children’s physical and mental health problems. Because of being subjected to violence, mothers often develop behavioral and mental problems, fail to establish an effective relationship with their children and may subject their children to violence. Such children tend to experience behavioral and emotional problems and show aggressive behaviors (38, 39). Because of the domestic violence their mothers experience, these children develop mood disorders (61). The indirect role of domestic violence committed against mothers in the formation of social and emotional developmental problems in children mediated by the stress experienced by the mothers is reported (22).

Our results showed a direct relationship between depression and children’s development. Maternal depression can entail negative consequences such as cognitive-behavioral problems for the children, because neuroplasticity is affected by the child’s contact with his caregiver and the stimulations he receives from the caregiver, which then affect the child’s long-term cognitive, emotional and social development (62). The first year of a child’s life is highly critical because brain development occurs during this period through the stimulations received from the caregiver and the communication established with the child. Depressed mothers fail to interact with their children or establish a mother-child bond (21). 

The mediating role of maternal depression in children’s development, especially in cases of chronic depression is reported (62). Feldman et al. reported a relationship between depression in mothers and emotional, social and behavioral problems in 9-month-old children (63).

Ali et al., also reported a relationship between maternal depression and children’s development in five domains (21). Vameghi et al. showed a relationship between maternal depression and development in 6-18 monthold children (16). Maternal depression during the first year after childbirth entails long-term complications and psychological problems for the children, as these children were 4 times more at risk for developing these problems (64).

The present study found that, mediated by depression, maternal anxiety is related indirectly to children’s development. Highly anxious mothers have higher levels of cortisol compared to mothers with lower anxiety scores and their children therefore experience greater behavioral and emotional problems (63). Ali et al. also reported a relationship between maternal anxiety and children’s development in five domains (21).

The results of the present study showed a direct relationship between maternal stress and children’s development. Infants of highly stressed women show restlessness, inattention to rules and high levels of behavioral problems at the age of two (65). Different sources also show a strong relationship between stressful events experienced by women and developmental delays in their children. Parents’ psychological problems appear to add to their stress and thus affect the relationship they establish with their children and thereby cause behavioral, psychological, and emotional problems in the children (26, 29). Children of mothers who have experienced high levels of stress during their pregnancy showed functional problems at the age of 5, although the mothers’ stress did not affect the children’s IQ (66). Stressful conditions in couples can affect their parenting behaviors and their own mental state and thus result in cognitive problems, irritation, reduced levels of energy and poor compliance with the children’s demands (67). The mother’s level of stress does not affect her child’s development at the age of two, but rather causes behavioral problems in the child at the age of four (68). Animal and human studies have reported long-term changes in the child’s cognitive, physical and behavioral outcomes mediated by the mother’s experience of physical and mental stress (69). 

Tang et al. showed the adverse effects of parental stress on development in 8 and 13 month-old children (70). 

**In conclusion, **children’s development is linked indirectly to domestic violence and anxiety (mediated by depression) and directly to depression and stress in the mother. The proposed model has a good fit.

Since mental problems in mothers, especially stress and depression, affect their own and their children’s physical and mental health and well-being, and since the anxiety formed through the mothers’ experience of domestic violence (as mediated by depression) can adversely affect their own and their children’s physical and mental health, maternal and child health professionals are recommended to prioritize the issue of maternal mental health and domestic violence against pregnant women and breastfeeding mothers in their design of preventive and supportive programs. 
